# The impact of changes to RS-1 on nursing management: a new approach to psychosocial risks in nursing

**DOI:** 10.1590/0034-7167.2025780501

**Published:** 2025-08-22

**Authors:** Sérgio Valverde Marques dos Santos, Zélia Marilda Rodrigues Resck, Vitória Cristina Silva

**Affiliations:** I Universidade Federal de Alfenas, Escola de Enfermagem. Alfenas, Minas Gerais, Brasil.

The recent update of Regulatory Standard 1 (RS-1), which establishes the general provisions and management of occupational risks, represents a significant transformation in the way psychosocial risks should be managed in the workplace. For nursing, a profession that is intrinsically linked to human care and that deals directly with situations of high emotional burden and stress, the changes introduced by RS-1 offer both challenges and opportunities to improve management practices and promote safer and healthier work environments.

Adopting the new guidelines requires a holistic and integrated view of the work environment, considering the multiple factors that affect professionals’ psychological well-being, such as excessive workload, interpersonal conflicts and exposure to situations of physical and psychological violence. The update of RS-1 reflects a growing concern for workers’ mental health, recognizing that effective management of these risks can not only improve work quality, but also nursing professionals’ health and satisfaction. According to the World Health Organization, “mental health is a state of well-being where an individual realizes their capabilities, copes with normal stresses, works productively, and contributes to their community”^(1)^.

RS-1’s new perspective on psychosocial risks has expanded the scope of the Risk Management Program (RMP), explicitly including psychosocial risks as part of the requirements for all organizations (Brazil, 2022). This change has a direct impact on health institutions, especially nursing workers, where psychosocial factors have always been a central concern. The introduction of specific guidelines for managing these risks demonstrates a clearer recognition that stress, emotional pressure, lack of social support and adverse working conditions can have devastating effects on professionals’ mental and physical health.

RS-1 requires healthcare managers, including nursing managers, to implement practices that actively address these risks, with the aim of mitigating the negative effects associated with stressful working conditions. Risk factors that should be monitored include task overload, shortage of human resources, insecurity in the workplace, and lack of recognition and emotional support^(2)^. For nursing workers, who are often exposed to these challenges, changing regulations bring not only an opportunity for development, but also a requirement for innovation in occupational health management practices. 

This new context brings challenges and opportunities for nursing management, since RS-1 implementation requires a profound transformation in risk management strategies within nursing services. It is not just about identifying psychosocial risks, but adopting a proactive and integrated approach to addressing them. For this transition to be successful, psychosocial risk management must be incorporated into daily routines, which implies a series of challenges and opportunities.

The first stage in implementing RS-1 is to conduct a thorough assessment of the psychosocial factors present in the work environment. This includes analyzing working conditions, the relationship between team members, and the emotional burden of the tasks performed. Identifying situations of excessive stress, moral harassment, physical and psychological violence, and other adverse factors is essential for an effective approach. According to RS-1, “the RMP must consider the psychosocial and ergonomic risks”^(2)^.

After assessment, it is necessary to adopt concrete strategies to mitigate the identified risks. This may include reorganizing nursing tasks to avoid work overload, creating adequate rest spaces, and offering psychological and emotional support. Moreover, fostering open communication and collaboration among team members can significantly reduce feelings of isolation and stress.

One of the main requirements of RS-1 is the implementation of a continuous system of monitoring and assessing workers’ mental health. This includes conducting regular check-ins, measuring stress indicators and analyzing the impact of the measures implemented. Creating an organizational culture of constant feedback helps to adjust practices and continually improve the work environment.

Another aspect to be worked on is the promotion of a culture of care. RS-1 also highlights the importance of an approach centered on care and well-being. In the context of nursing, this involves not only caring for patients, but also for professionals. Creating an environment in which mental health is valued and where workers feel safe to express their concerns and seek help is essential to prevent burnout and other conditions associated with occupational stress.

The development of leadership skills, effective communication, conflict management and problem-solving becomes essential. In addition, the implementation of policies and practices that promote mental health must be a daily commitment, reflecting a cultural change within the organization. Nursing managers need to be an advocate for the team’s emotional well-being, recognizing that nursing professionals’ mental health directly impacts care quality^(3-4)^.

In this context, managers play a central role in implementing the changes imposed by RS-1. To be effective in this process, they need not only to understand the standard’s new requirements, but also to have a strategic and humanized vision of people management. They must act as a facilitator, ensuring that the resources necessary for implementing RS-1 are available and that the team is trained and made aware of psychosocial risks.

The implementation of changes to RS-1 represents a significant advance in the management of psychosocial risks in the nursing workplace. For these advances to translate into concrete improvements, it is essential to adopt a comprehensive approach that involves the identification, prevention and constant monitoring of the factors that affect workers’ mental health. By integrating psychosocial risk management into their daily practice, nursing managers not only comply with the standard, but also contribute to the creation of a healthier, safer and more sustainable work environment for all nursing professionals.
