# Supplementary material for: The impact of changes to RS-1 on nursing management: a new approach to psychosocial risks in nursing
Source: Rev Bras Enferm. 2025 Aug 22;78(5):e780501. doi: 10.1590/0034-7167.2025780501 (PMC12380419; doi:10.1590/0034-7167.2025780501)
Supplement: Translation

# Translation: O impacto das alterações da NR-1 na gestão em enfermagem: uma nova abordagem para os riscos psicossociais na enfermagem

**DOI:** 10.1590/0034-7167.2025780501pt

**Published:** 2025-07-27

**Authors:** Sérgio Valverde Marques dos Santos, Zélia Marilda Rodrigues Resck, Vitória Cristina Silva

**Affiliations:** I Universidade Federal de Alfenas, Escola de Enfermagem. Alfenas, Minas Gerais, Brasil.

A recente atualização da Norma Regulamentadora nº 1 (NR-1), que estabelece as disposições gerais e o gerenciamento de riscos ocupacionais, representa uma transformação significativa na maneira como os riscos psicossociais devem ser gerenciados no ambiente de trabalho. Para a enfermagem, profissão que está intrinsecamente ligada ao cuidado humano e que lida diretamente com situações de alta carga emocional e estresse, as mudanças introduzidas pela NR-1 oferecem tanto desafios quanto oportunidades de aprimorar as práticas de gestão e promover ambientes de trabalho mais seguros e saudáveis.

A adoção das novas diretrizes exige uma visão holística e integrada do ambiente de trabalho, considerando os múltiplos fatores que afetam o bem-estar psicológico dos profissionais, como a carga de trabalho excessiva, os conflitos interpessoais e a exposição às situações de violência física e psicológica. A atualização da NR-1 reflete uma preocupação crescente com a saúde mental dos trabalhadores, reconhecendo que a gestão eficaz desses riscos não apenas pode melhorar a qualidade do trabalho, mas também a saúde e a satisfação dos profissionais de enfermagem. Segundo a Organização Mundial da Saúde, “a saúde mental é um estado de bem-estar em que o indivíduo realiza suas capacidades, pode fazer frente ao estresse normal da vida, trabalhar de forma produtiva e frutífera e contribuir para sua comunidade”^(1)^.

A nova perspectiva da NR-1 sobre riscos psicossociais ampliou o escopo do Programa de Gerenciamento de Riscos (PGR), incluindo, explicitamente, os riscos psicossociais como parte das exigências para todas as organizações (Brasil, 2022). Esta alteração tem um impacto direto nas instituições de saúde, especialmente nos trabalhadores de enfermagem, em que os fatores psicossociais sempre foram uma preocupação central. A introdução de diretrizes específicas para a gestão desses riscos demonstra um reconhecimento mais claro que o estresse, a pressão emocional, a falta de apoio social e as condições de trabalho adversas podem ter efeitos devastadores na saúde mental e física dos profissionais.

A NR-1 exige que os gestores de saúde, incluindo os de enfermagem, implementem práticas que abordem ativamente esses riscos, com o objetivo de mitigar os efeitos negativos associados às condições de trabalho estressantes. Entre os fatores de risco que devem ser monitorados, destacam-se a sobrecarga de tarefas, a escassez de recursos humanos, a insegurança no ambiente de trabalho, e a falta de reconhecimento e suporte emocional^(2)^. Para os trabalhadores de enfermagem, frequentemente expostos a esses desafios, a mudança nas regulamentações traz não apenas uma oportunidade de desenvolvimento, mas também uma exigência de inovação nas práticas de gerenciamento de saúde ocupacional. 

Esse novo contexto traz desafios e oportunidades para a gestão em enfermagem, pois a implementação da NR-1 requer uma transformação profunda nas estratégias de manejo de riscos dentro dos serviços de enfermagem. Não se trata apenas de identificar os riscos psicossociais, mas de adotar uma abordagem proativa e integrada para enfrentá-los. Para que essa transição seja bem-sucedida, deve haver a incorporação da gestão dos riscos psicossociais nas rotinas diárias, o que implica uma série de desafios e oportunidades.

A primeira etapa na implementação da NR-1 é realizar uma avaliação minuciosa dos fatores psicossociais presentes no ambiente de trabalho. Isso inclui a análise das condições de trabalho, da relação entre os membros da equipe e da carga emocional das tarefas desempenhadas. A identificação de situações de estresse excessivo, assédio moral, violência física e psicológica, e outros fatores adversos é fundamental para uma abordagem eficaz. Segundo a NR-1, “o PGR deve considerar os riscos psicossociais e ergonômicos”^(2)^.

Após a avaliação, é necessário adotar estratégias concretas para mitigar os riscos identificados. Isso pode incluir a reorganização das tarefas de enfermagem para evitar a sobrecarga de trabalho, a criação de espaços de descanso adequados e a oferta de apoio psicológico e emocional. Além disso, fomentar a comunicação aberta e a colaboração entre os membros da equipe pode reduzir significativamente a sensação de isolamento e estresse.

Uma das principais exigências da NR-1 é a implementação de um sistema contínuo de monitoramento e avaliação da saúde mental dos trabalhadores. Isso inclui a realização de *check-ins* regulares, a medição de indicadores de estresse e a análise do impacto das medidas implementadas. A criação de uma cultura organizacional de *feedback* constante ajuda a ajustar as práticas e melhorar continuamente o ambiente de trabalho.

Um outro aspecto a ser trabalhado é a promoção de uma cultura de cuidado. A NR-1 também destaca a importância de uma abordagem centrada no cuidado e no bem-estar. No contexto da enfermagem, isso envolve não apenas o cuidado com os pacientes, mas também com os profissionais. A criação de um ambiente em que a saúde mental seja valorizada e onde os trabalhadores se sintam seguros para expressar suas preocupações e buscar ajuda é essencial para prevenir o *burnout* e outras condições associadas ao estresse ocupacional.

O desenvolvimento de habilidades de liderança, comunicação eficaz, gestão de conflitos e resolução de problemas torna-se essencial. Além disso, a implementação de políticas e práticas que promovam a saúde mental deve ser um compromisso diário, refletindo uma mudança cultural dentro da organização. O gestor de enfermagem precisa ser um defensor do bem-estar emocional da equipe, reconhecendo que a saúde mental dos profissionais de enfermagem impacta diretamente a qualidade do cuidado^(3-4)^.

Nesse contexto, o gestor ocupa um papel central na implementação das mudanças impostas pela NR-1. Para ser eficaz nesse processo, precisa não apenas compreender as novas exigências da norma, mas também ter uma visão estratégica e humanizada sobre a gestão de pessoas. Ele deve atuar como um facilitador, assegurando que os recursos necessários para a implementação da NR-1 estejam disponíveis e que a equipe seja treinada e sensibilizada em relação aos riscos psicossociais.

A implementação das alterações da NR-1 representa um avanço significativo na gestão de riscos psicossociais no ambiente de trabalho da enfermagem. Para que esses avanços se traduzam em melhorias concretas, é essencial que seja adotada uma abordagem abrangente, que envolva a identificação, prevenção e monitoramento constante dos fatores que afetam a saúde mental dos trabalhadores. Ao integrar a gestão de riscos psicossociais em sua prática diária, os gestores de enfermagem não apenas cumprem a norma, mas também contribuem para a criação de um ambiente de trabalho mais saudável, seguro e sustentável para todos os profissionais de enfermagem.

# Translation: El impacto de los cambios en la NR-1 en la gestión de enfermería: un nuevo enfoque de los riesgos psicosociales en enfermería

**DOI:** 10.1590/0034-7167.2025780501es

**Published:** 2025-07-27

**Authors:** Sérgio Valverde Marques dos Santos, Zélia Marilda Rodrigues Resck, Vitória Cristina Silva

**Affiliations:** I Universidade Federal de Alfenas, Escola de Enfermagem. Alfenas, Minas Gerais, Brasil.

La reciente actualización de la Norma Regulatoria n° 1 (NR-1), que establece las disposiciones generales y la gestión de los riesgos laborales, representa una transformación significativa en la gestión de los riesgos psicosociales en el entorno laboral. Para la enfermería, profesión intrínsecamente vinculada al cuidado humano y que se ocupa directamente de situaciones de alta carga emocional y estrés, los cambios introducidos por la NR-1 ofrecen tanto desafíos como oportunidades para mejorar las prácticas de gestión y promover entornos laborales más seguros y saludables.

La adopción de las nuevas directrices requiere una visión holística e integrada del entorno laboral, considerando los múltiples factores que afectan el bienestar psicológico de los profesionales, como la sobrecarga de trabajo, los conflictos interpersonales y la exposición a situaciones de violencia física y psicológica. La actualización de la NR-1 refleja una creciente preocupación por la salud mental de los trabajadores, reconociendo que la gestión eficaz de estos riesgos no solo puede mejorar la calidad del trabajo, sino también la salud y la satisfacción de los profesionales de enfermería. Según la Organización Mundial de la Salud, “la salud mental es un estado de bienestar en el que el individuo es consciente de sus capacidades, puede afrontar las tensiones normales de la vida, trabajar de forma productiva y fructífera y contribuir a su comunidad”^(1)^.

La nueva perspectiva del NR-1 sobre los riesgos psicosociales ha ampliado el alcance del Programa de Gestión de Riesgos (PGR), incluyendo explícitamente los riesgos psicosociales como parte de los requisitos para todas las organizaciones (Brasil, 2022). Este cambio tiene un impacto directo en las instituciones de salud, especialmente en el personal de enfermería, donde los factores psicosociales siempre han sido una preocupación central. La introducción de directrices específicas para la gestión de estos riesgos demuestra un reconocimiento más claro de que el estrés, la presión emocional, la falta de apoyo social y las condiciones laborales adversas pueden tener efectos devastadores en la salud mental y física de los profesionales.

La NR-1 exige que los gerentes de atención médica, incluyendo los gerentes de enfermería, implementen prácticas que aborden activamente estos riesgos, con el fin de mitigar los efectos negativos asociados a las condiciones laborales estresantes. Los factores de riesgo que deben monitorearse incluyen la sobrecarga de trabajo, la escasez de recursos humanos, la inseguridad laboral y la falta de reconocimiento y apoyo emocional^(2)^. Para los trabajadores de enfermería, que a menudo están expuestos a estos desafíos, el cambio de regulaciones trae no sólo una oportunidad de desarrollo, sino también un requisito de innovación en las prácticas de gestión de la salud ocupacional. 

Este nuevo contexto presenta desafíos y oportunidades para la gestión de enfermería, ya que la implementación de la NR-1 requiere una profunda transformación en las estrategias de gestión de riesgos dentro de los servicios de enfermería. No se trata solo de identificar los riesgos psicosociales, sino de adoptar un enfoque proactivo e integrado para abordarlos. Para que esta transición sea exitosa, la gestión de riesgos psicosociales debe incorporarse en la rutina diaria, lo que implica una serie de desafíos y oportunidades.

El primer paso para implementar la NR-1 es realizar una evaluación exhaustiva de los factores psicosociales presentes en el entorno laboral. Esto incluye el análisis de las condiciones laborales, las relaciones entre los miembros del equipo y la carga emocional de las tareas desempeñadas. Identificar situaciones de estrés excesivo, acoso moral, violencia física y psicológica, y otros factores adversos es esencial para un abordaje eficaz. Según la NR-1, “la PGR debe considerar los riesgos psicosociales y ergonómicos”^(2)^.

Tras la evaluación, es necesario adoptar estrategias concretas para mitigar los riesgos identificados. Esto puede incluir la reorganización de las tareas de enfermería para evitar la sobrecarga laboral, la creación de espacios de descanso adecuados y el apoyo psicológico y emocional. Además, fomentar la comunicación abierta y la colaboración entre los miembros del equipo puede reducir significativamente la sensación de aislamiento y estrés.

Uno de los principales requisitos de la NR-1 es la implementación de un sistema continuo de monitoreo y evaluación de la salud mental de los trabajadores. Esto incluye la realización de controles periódicos, la medición de indicadores de estrés y el análisis del impacto de las medidas implementadas. Crear una cultura organizacional de retroalimentación constante facilita el ajuste de las prácticas y la mejora continua del ambiente laboral.

Otro aspecto a trabajar es la promoción de una cultura del cuidado. El NR-1 también destaca la importancia de un enfoque centrado en el cuidado y el bienestar. En el contexto de la enfermería, esto implica no solo la atención a los pacientes, sino también a los profesionales. Crear un entorno donde se valore la salud mental y donde los trabajadores se sientan seguros para expresar sus inquietudes y buscar ayuda es esencial para prevenir el síndrome de *burnout* y otras afecciones asociadas al estrés laboral.

El desarrollo de habilidades de liderazgo, comunicación efectiva, gestión de conflictos y resolución de problemas se vuelve esencial. Además, la implementación de políticas y prácticas que promuevan la salud mental debe ser un compromiso diario, reflejando un cambio cultural dentro de la organización. El gerente de enfermería debe ser un defensor del bienestar emocional del equipo, reconociendo que la salud mental de los profesionales de enfermería impacta directamente en la calidad de la atención^(3-4)^.

En este contexto, el gerente desempeña un papel fundamental en la implementación de los cambios impuestos por la NR-1. Para ser eficaz en este proceso, no solo debe comprender los nuevos requisitos de la norma, sino también tener una visión estratégica y humanizada de la gestión de personas. Debe actuar como facilitador, garantizando la disponibilidad de los recursos necesarios para la implementación de la NR-1 y que el equipo esté capacitado y sensibilizado sobre los riesgos psicosociales.

La implementación de los cambios a la NR-1 representa un avance significativo en la gestión de riesgos psicosociales en el entorno laboral de enfermería. Para que estos avances se traduzcan en mejoras concretas, es fundamental adoptar un enfoque integral que incluya la identificación, la prevención y el seguimiento constante de los factores que afectan la salud mental de los trabajadores. Al integrar la gestión de riesgos psicosociales en su práctica diaria, los directivos de enfermería no solo cumplen con la norma, sino que también contribuyen a la creación de un entorno laboral más saludable, seguro y sostenible para todos los profesionales de enfermería.
